# Victoria Park Consumption Hospital

**Published:** 1889-01-26

**Authors:** T. Storrar Smith


### EDITOR'S LETTER-BOX.

[Our Correspondents are reminded that prolixity is a great bar to publication, and that brevity of style and conciseness of statement greatly facilitate early insertion.]

### VICTORIA PARK CONSUMPTION HOSPITAL.

MR. T. STORRAR SMITH, Secretary to the City of London Hospital for Diseases of the Chest, Victoria Park, E., writes: The appeal inserted in your issue of the 29th ult. on behalf of this hospital has come under the notice of the committee, and, while they are desirous of acknowledging your kind interest in the institution and of thanking you for bringing it before your readers, they also wish to remove from your mind some erroneous impressions which you appear to have received in reference to this hospital. First, I would assure you that we are ready at all times to take advantage of any opportunity afforded by your excellent journal, or by any other organ of the press, in making known our work and our requirements. I much regret that I was not aware of your intention of issuing these appeals, or I would gladly have furnished you with accurate and suitable information as regards this hospital. Then, as to advertising: you have been misinformed on this point. We do advertise, with due regard to economy, at such times and in such mediums as is considered likely to be most advantageous to the institution. Economy in this respect is as necessary as in any other item of expenditure, and the number of our advertisements must be regulated by the amount which the committee think they are justified in expending on this account. Perhaps you will allow me to correct one other statement made in the appeal. Our deficit for 1887 is there stated as £3,000. The actual excess of expenditure over income was £1,720—fortunately not so large as you suppose, but so serious as to render imperative the exercise of care in those branches of expenditure which cannot be regarded as absolutely necessary.

